# Significance of Vertigo, Imbalance, and Other Minor Symptoms in Hyperacute Treatment of Posterior Circulation Stroke

**DOI:** 10.3389/fneur.2022.845707

**Published:** 2022-05-16

**Authors:** Min Kim, So Young Park, Sung Eun Lee, Jin Soo Lee, Ji Man Hong, Seong-Joon Lee

**Affiliations:** ^1^Department of Neurology, Ajou University Medical Center, Ajou University School of Medicine, Suwon, South Korea; ^2^Department of Emergency Medicine, Ajou University Medical Center, Ajou University School of Medicine, Suwon, South Korea

**Keywords:** posterior circulation ischemic stroke, thrombolysis code, thrombolysis, vertigo, disequilibrium

## Abstract

**Background:**

This study aimed to determine the clinical significance of acute vestibular syndrome (AVS)/acute imbalance syndrome (AIS) in posterior circulation stroke (PCS) and how it should be addressed in the thrombolysis code.

**Methods:**

Our institution has recently changed its thrombolysis code from one that is generous to AVS/AIS to one that is exclusive. The subjects in this study were patients with PCS who presented before this transition (May 2016 to April 2018, period 1) and those who presented after (January 2019 to December 2020, period 2) with an onset-to-door time of 4.5 h. Hyperacute stroke treatment was compared between the two periods. The clinical significance of AVS/AIS was evaluated by dichotomizing the patients' clinical severity to minor or major deficits, then evaluating the significance of AVS/AIS in each group. Presenting symptoms of decreased mental alertness, hemiparesis, aphasia (anarthria), or hemianopsia were considered major PCS symptoms, and patients who did not present with these symptoms were considered minor PCS.

**Results:**

In total, 114 patients presented in period 1 and 114 in period 2. Although the code activation rate was significantly lower in period 2 (72.8% vs. 59.7%), *p* = 0.04, there were no between-group differences in functional outcomes (mRS score at 3 months; 1 [0–3] vs. 0 [0–3], *p* = 0.18). In 77 patients with PCS and AVS/AIS, the difference in code activation rate was not significant according to changes in thrombolysis code. In minor PCS, AVS/AIS was associated with lower NIHSS scores, lower early neurological deterioration rates, and favorable outcomes. In major PCS, while AVS/AIS was not associated with outcomes, the majority of cases were prodromal AVS/AIS which simple vertigo and imbalance symptoms were followed by a major PCS symptom.

**Conclusions:**

This study failed to show differences in outcome in patients with PCS according to how AVS/AIS is addressed in the stroke thrombolysis code. In patients with minor PCS, AVS/AIS was associated with a benign clinical course. Prompt identification of prodromal AVS/AIS is essential.

## Introduction

About 12–19% of all strokes treated by intravenous thrombolysis (IVT) are posterior circulation stroke (PCS) (1, 2), and ~10% of large vessel occlusion strokes involve the vertebrobasilar artery (3). However, clinical evidence of hyperacute stroke treatment in the posterior circulation lags behind anterior circulation strokes. The differences in safety and outcomes of thrombolysis between the anterior and posterior circulation is continuously being investigated (4) while the treatment effect of mechanical thrombectomy is still debated in the posterior circulation (3, 5, 6). Two randomized trials failed to prove benefit for endovascular therapy compared to medical therapy in patients with vertebrobasilar occlusion (3, 5), while one non-randomized cohort study showed better functional outcomes and reduced mortality (6). Hyperacute treatment of PCS is complicated by a number of reasons.

First, patients with PCS differ from those with anterior circulation ischemic stroke (ACS) in terms of presenting symptoms and signs, contributing to delay in diagnosis (7). A key symptom is dizziness and vertigo. Acute dizziness accounts for 3.5–11% of all patient visits to the emergency department (ED) (8, 9). Stroke accounts for 3–5% of all presentations to the ED with vertigo and dizziness (10, 11), while symptoms of dizziness or vertigo occur in 47–75% of patients with PCS (12). Accordingly, PCS patients may present as acute vestibular syndrome (AVS: vertigo, nystagmus, nausea/vomiting) or as acute imbalance syndrome (AIS: dizziness, sudden unsteadiness of stance and/or gait, no nystagmus) (13). Identification of stroke in AVS/AIS is a diagnostic challenge. While there have been numerous studies focusing on identification of central vertigo, it is still not appropriately addressed, resulting in misdiagnosis (11, 14).

Second, the symptoms of PCS is under-represented in scales evaluating stroke severity (15). The National Institutes of Health Stroke Scale (NIHSS) is the tool most widely used to evaluate the severity of acute stroke and focuses on the status of the anterior circulation. However, symptoms of PCS such as vertigo, nystagmus, and truncal ataxia, which are components of AVS/AIS are not fully included in the NIHSS, which complicates thrombolysis for PCS (16). Moreover, According to a previous study, the prognosis may be poorer in patients with PCS than in those with ACS, even with a low NIHSS score of ≤4 (17). Furthermore, according to the National Institute of Neurological Disorders and Stroke criteria, symptoms that arise in the brainstem, such as vertigo, dizziness, and dysarthria, are not defined as transient ischemic attacks (18). Therefore, the clinical severity of PCS may be underrated.

Stroke centers capable of providing thrombolysis and endovascular reperfusion treatment implement critical pathway and formal protocols to accelerate the delivery of hyperacute stroke treatments (19). Thus, when a patient suspected of stroke arrives to the ED, emergency medicine physicians or emergency nurses are able to activate the institutional thrombolysis code, calling for stroke physicians to determine thrombolysis eligibility, while enabling neurointerventional team members to prepare for possible endovascular thrombectomy (20). How the institutional thrombolysis code addresses acute vertigo/imbalance may have significant influences on hyperacute management of PCS, and according clinical outcomes of this population. Considering that only 3–5% of stroke patients present with dizziness, indiscriminate referrals and code activation will result in unnecessary costs and wasted manpower (10, 11, 21). On the contrary, neglection of AVS/AIS in the thrombolysis code may result in neglected treatment and resultant early neurological deterioration (END).

Recently, we had the opportunity to address such concerns, for we have recently modified our thrombolysis code from one that is generous to AVS/AIS to a more strict code that focuses on major deficits. We hypothesized that modifications in thrombolysis code to one that is exclusive to AVS/AIS will be detrimental to PCS stroke outcomes by causing delays in critical treatment. Such influence on outcome may differ according to whether the patient presented with AVS/AIS or not, and also if the patient presented with accompanying major neurological deficits, which would justify thrombolysis by itself, or minor neurological deficits. To confirm this hypothesis, using the institutional posterior circulation stroke database, we compared clinical outcomes in patients with PCS according to changes in stroke thrombolysis code. Using the same population, we also aimed to evaluate the clinical significance of AVS/AIS in PCS patients after dividing the patients to minor and major neurological deficits. Through this analysis, we further sought to identify patients that are likely to deteriorate or result in poor outcomes, who might benefit through faster and more proactive treatment.

## Methods

### Study Participants and Changes to the Thrombolysis Activation Code

This retrospective, single-center, observational study was approved by the Institutional Review Board of Ajou University Hospital, Suwon, Korea and was conducted in accordance with the Declaration of Helsinki (AJIRB-MED-MDB-21-613). The requirement for informed consent was waived in view of the retrospective nature of the research.

Patient data were obtained from our institutional stroke registry, which contains information on all patients admitted with ischemic stroke. The institutional stroke thrombolysis code was modified in April 2018 to reflect the relative importance of the thrombolysis code to detect large vessel occlusion strokes (22), and to prepare for increases in thrombolysis code activation triggered by success of the late window thrombectomy trials (23, 24), aiming for efficient use of resources and manpower in our hospital. Before then, a thrombolysis code based on “sudden, side, symptoms” with more permissive symptomatology was utilized. “Sudden” was classified as an abrupt neurological deficit within 6 h from onset to arrival in the emergency room (ER). “Side” was defined as unilateral weakness affecting the face, an arm, or a leg. “Symptoms” that activated the thrombolysis code were as follows: difficulty walking, dysarthria or aphasia, motor weakness, abnormal behavior, sensory changes, visual disturbance, or others, in which vertigo could be incorporated (Table 1). After May 2018 (period 2), we used a thrombolysis code that focused on major deficits and prohibited activation for isolated vertigo or disequilibrium. This thrombolysis code used the modified Face Arm Speech Time test (25). Relevant indications for activation of the thrombolysis code were two or more of consecutive unilateral hemiplegia of the face, arms, and legs, aphasia, and loss of consciousness without other clear causes within 8 h from onset to arrival in the ER. Symptoms of mono limb paresis, bilateral paresis without change in mental status, amnesia, vertigo, disequilibrium, and sensory change were contraindications to activation of the thrombolysis code (Table 1). In this study, data for all PCS patients who presented to the ED within 4.5 h of onset of symptoms between May 2016 and April 2018 (period 1) or between January 2019 and December 2020 (period 2) were analyzed. The diagnosis of PCS was confirmed by MRI. Stroke lesions were classified into medulla, pons, midbrain, anterior inferior cerebellar artery, posterior inferior cerebellar artery, superior cerebellar artery, thalamus, and posterior cerebral artery lesions excluding thalamus, in which diffusion restriction was confirmed on MRI. Patients with simultaneous PCS and anterior circulation stroke were excluded. The 8 months in between was considered a transitional period and not included in the analysis. In Period 1, 1,592 patients were hospitalized for ischemic stroke, of which 457 had PCS. Of the patients with PCS, 114 patients with PCS visited the ED within 4.5 h of symptoms. Likewise, in period 2, 1,508 patients were hospitalized for ischemic stroke, and among them, 475 patients with PCS were admitted for ED. Among them, 114 patients who visited to ED within 4.5 h of symptom onset were enrolled in the study in period 2.

**Table 1:** Thrombolysis code according to study periods.

**Study period**	**Model**	
Period 1 (May 2016 to April 2018)	3S cube model (Sudden, Side, Symptom)	
	Sudden	Within 6 h
	Side	Unilateral weakness in an arm, a leg, or the face
	Symptom	1) Gait difficulty 2) Dysarthria or aphasia 3) Motor weakness 4) Abnormal behavior 5) Sensory change 6) Visual disturbance 7) Other (including vertigo and disequilibrium)
Period 2 (January 2019 to December 2020)	FAST model	
	Time	Within 8 h
	Symptom	1) Unilateral hemiplegia in an arm, a leg, or the face 2) Aphasia 3) Loss of consciousness without clear causes
	Exclusion	1) Weakness in one limb 2) Bilateral paresis without change in mental status 3) Amnesia 4) Vertigo 5) Disequilibrium 6) Sensory changes

*Period 1: Thrombolysis code focusing on “sudden, side, symptoms” with more permissive symptomatology (May 2016 to April 2018) Period 2: Thrombolysis code focusing on major deficits and prohibited its activation for isolated vertigo or disequilibrium (January 2019 to December 2020)*.

### Clinical Variables and Classification of Major and Minor PCS

Patient data regarding hyperacute management of stroke such as onset to visit time, code activation rate, door to neurologist referral time, median NIHSS scores, reperfusion treatment rate, END rate, and neurological outcomes were collected. According to the TOAST classification, the etiology of stroke was classified as large artery atherosclerosis, cardioembolism, small vessel occlusion, stroke of other determined etiology, or stroke of undetermined etiology (26).

Reperfusion therapy was defined as endovascular treatment (EVT) or IVT. Symptoms were confirmed through the NIHSS recorded in the medical record. Presenting symptoms of decreased mental alertness (defined as a level of consciousness subset score of ≥1 on the NIHSS), hemiparesis involving at least two compartments (face, upper limb, lower limb), aphasia (anarthria), or hemianopsia were considered major neurological deficits and patients with according deficits were termed major PCS, and patients who did not present with these symptoms were considered to present with minor deficits, and termed minor PCS (13). This classification was based on the idea that major deficits are usually regarded clearly disabling, and justify reperfusion treatments in itself, while there may be considerable uncertainty for AVS/AIS accompanied by minor deficits, and management of this subgroup would more likely be influenced by changes in thrombolysis code. END was defined as an increase of two or more points from the initial NIHSS after admission.

In patients with minor PCS, we defined an unfavorable outcome as that corresponding to a modified Rankin Scale (mRS) score of 2–6 at 3 months or END during hospitalization and an excellent outcome indicated by a mRS score of 0–1 and no END. In patients with major PCS, a good outcome was defined as a mRS score of 0–2 and a poor outcome as a mRS score of 3–6 at 3 months.

### Evaluation of Vertigo, Imbalance, and Central Oculomotor Findings

The electronic medical records were retrospectively reviewed to identify PCS patients who presented with vertigo or imbalance. These symptoms were subclassified as follows: AVS (vertigo, nausea, vomiting, with or without spontaneous nystagmus, and continuously present at the time of ED presentation); acute imbalance syndrome (AIS, acute onset of unsteadiness in stance and gait that persisted at ED presentation without spontaneous nystagmus); transient AVS/AIS (resolution of symptoms before presentation to the ER); or prodromal AVS/AIS (vertigo or imbalance followed by major neurological deficits [as classified in the above section] that led to presentation to the ER). Horizontal/vertical gaze-evoked nystagmus, vertical or purely torsional spontaneous nystagmus, or ophthalmoparesis (27) were considered central oculomotor signs. PCS patients without symptoms of vertigo or imbalance were subclassified as non-AVS/AIS group. Our institution routinely performs CT angiography to patients with neurological deficits suspected of stroke, and also to patients that present with AVS/AIS. For the analysis on the relationship between stenosis or occlusion and prognosis, vertebrobasilar steno-occlusion was defined as the presence of significant occlusion or stenosis of more than 50% in the basilar artery or both vertebral arteries (28–30).

### Statistical Analysis

For the first part of the study, the total PCS population was dichotomized according to changes in thrombolysis code as patients that presented in period 1 and those that presented in period 2 (Table 1). The two groups were compared to clarify whether a change in the thrombolysis code that excludes minor neurological deficits such as vertigo resulted in delays in treatment and changes in outcome. This analysis was also performed in the subgroup of patients with PSC who presented with AVS/AIS, and subgroup of patients that presented with minor PCS. Next, the clinical significance of AVS/AIS in PCS was evaluated. Given the likelihood that obvious major neurological deficits may be more disabling for the patient and influence clinical triage, the patients were divided according to whether PCS was major or minor. Univariate and multivariate analyses were performed to clarify the significance of vertigo and acute imbalance on the clinical characteristics and prognosis of minor and major PCS.

Continuous variables are summarized as the mean ± standard deviation, number (percentage), or median (interquartile range) and categorical variables as counts and percentages as appropriate. For comparing two groups, Continuous variables were compared using the Student's *t*-test and Mann-Whitney *U* test and categorical variables using the chi-square test. For comparison of three groups, continuous variables were compared using one-way ANOVA and *post hoc* Tamhane's test, and categorical variables by using the chi-square test. Multiple logistic regression was performed to identify predictors of outcome, including clinically relevant variables. Associations are presented as the odds ratio (OR) with the corresponding 95% confidence interval (CI). Statistical analyses were performed using R statistical software (version 3.6.3; R Foundation for Statistical Computing, Vienna, Austria) and the IBM SPSS package (version 25.0 for Windows; IBM Corp., Armonk, NY, USA). A *p*-value of <0.05 was considered statistically significant.

## Results

### Differences in Code Activation and Treatment Outcomes According to Changes in Stroke Code

Of 228 patients diagnosed to have PCS between May 2016 and December 2020, 114 presented in period 1 (age, 65 ± 14 years; male, 69 [60.5%]) and 114 presented in period 2 (age, 67 ± 12 years; male, 74 [64.9%]). The rates of thrombolysis code activation were significantly lower in period 2 (72.8% vs. 59.7%, *p* = 0.04). However, there was no significant difference in time to door (111 ± 66 min vs. 126 ± 66 min, *p* = 0.10), ED door to neurology department referral time (51 ± 123 min vs. 75 ± 95 min, *p* = 0.11), median initial NIHSS score (3 [1–13] vs. 5 [2–10], *p* = 0.44), intravenous thrombolysis rate (21.1% vs. 17.0%, *p* = 0.54), and frequency of EVT (22.8% vs. 15.0%, *p* = 0.19). Also, there was no significant difference in door to needle time (53 ± 22 min vs. 74 ± 48, *p* = 0.10) and door to groin puncture time (120 ± 38 min vs. 154 ± 162, *p* = 0.40). Furthermore, there was no difference in the frequency of a mRS score of 0–1 (57.0% vs. 64.0%, *p* = 0.34) or 0–2 (64.9% vs. 71.9%, *p* = 0.32) at 3 months, or frequency of END (17.5% vs. 16.7%, *p* > 0.99) between the two periods (Table 2).

**Table 2:** Demographics, hyperacute treatments, and dizziness classification of enrolled patients.

	**All patients (*****n** **=*** **228)**			**AVS/AIS (***n* = **77)**			**Minor PCS (*n =* 96)**		
	**Period 1**	**Period 2**	***p*-value**	**Period 1**	**Period 2**	***p*-value**	**Period 1**	**Period 2**	***p*-value**
	**(*n =* 114)**	**(*n =* 114)**		**(*n =* 39)**	**(*n =* 38)**		**(*n =* 53)**	**(*n =* 43)**	
Age (years)	65 ± 14	67 ± 12	0.30	61 ± 15	65 ± 13	0.21	63 ± 13	63 ± 11	0.86
Sex (male, %)	69 (60.5%)	74 (64.9%)	0.58	28 (71.8%)	26 (68.4%)	0.94	37 (69.8%)	30 (69.8%)	>0.99
Onset to visit time (min)	111 ± 66	126 ± 66	0.10	115 ± 66	124 ± 57	0.53	122 ± 67	142 ± 70	0.16
Door to neurology department referral time (min)	51 ± 123	75 ± 95	0.11	104 ± 193	128 ± 115	0.50	80 ± 169	119 ± 107	0.17
Code activation, *n* (%)	83 (72.8%)	68 (59.7%)	0.04	20 (51.3%)	17 (44.7%)	0.73	31 (58.5%)	12 (27.9%)	0.005
IVT, *n* (%)	24 (21.1%)	19 (17.0%)	0.54	5 (12.8%)	4 (11.1%)	>0.99	2 (3.8%)	0 (0.0%)	0.57
Door to needle time (min)	53 ± 22	74 ± 48	0.10	73 ± 37	67 ± 41	0.82	46 ± 0.71	–	
EVT, *n* (%)	26 (22.8%)	17 (15.0%)	0.19	7 (18.0%)	6 (16.2%)	>0.99	1 (1.9%)	1(2.3%)	>0.99
Door to groin puncture time (min)	120 ± 38	154 ± 162	0.40	134 ± 49	214 ± 276	0.52	95	60	–
Initial NIHSS, median	3 [1–13]	5 [2–10]	0.44	2 [1–6]	2 [1–5]	0.95	1 [0–3]	2 [0.5–2]	0.73
3 months mRS, median	1 [0–3]	1 [0–3]	0.18	1 [0–2.5]	1 [0–1]	0.34	1 [0–1]	0 [0–1]	0.09
3 months mRS 0–1, n (%)	65 (57.0%)	73 (64.0%)	0.34	25 (64.1%)	29 (76.3%)	0.36	45 (84.9%)	41 (95.4%)	0.18
3 months mRS 0–2, *n* (%)	74 (64.9%)	82 (71.9%)	0.32	29 (74.4%)	31 (81.6%)	0.62	47 (88.7%)	41 (95.4%)	0.42
END, *n* (%)	20 (17.5%)	19 (16.7%)	>0.99	4 (10.3%)	6 (15.8%)	0.70	6 (11.3%)	4 (9.3%)	>0.99
**Dizziness classification**			0.24			0.16			0.46
None, *n* (%)	75 (65.8%)	76 (66.7%)		0 (0.0%)	0 (0.0%)		31 (58.5%)	20 (46.5%)	
AVS, *n* (%)	11 (9.7%)	14 (12.3%)		11 (28.2%)	14 (36.8%)		10 (18.9%)	11 (25.6%)	
AIS, *n* (%)	9 (7.9%)	13 (11.4%)		9 (23.1%)	13 (34.2%)		7 (13.2%)	10 (23.3%)	
Prodromal AVS, *n* (%)	15 (13.2%)	7 (6.1%)		15 (38.5%)	7 (18.4%)		1 (1.9%)	0 (0.0%)	
Prodromal AIS, *n* (%)	0 (0.0%)	2 (1.8%)		0 (0.0%)	2 (5.3%)		0 (0.0%)	0 (0.0%)	
Transient AVS/AIS, *n* (%)	4 (3.5%)	2 (1.8%)		4 (10.3%)	2 (5.3%)		4 (7.6%)	2 (4.7%)	

*AIS, acute imbalance syndrome; AVS, acute vestibular syndrome; END, early neurological deterioration; EVT, endovascular treatment; IVT, intravenous thrombolysis; NIHSS, National Institutes of Health Stroke Scale; mRS, modified Rankin Scale; PCS, posterior circulation ischemic stroke*.

Accompanying symptoms of acute vertigo or imbalance syndrome were present in 39 patients (34.2%) during period 1 and in 38 (33.3%) during period 2. Analysis of the AVS/AIS group according to time period did not reveal any significant difference in patient age (61 ± 15 years vs. 65 ± 13 years, *p* = 0.21), sex distribution (male, 28 [71.8%] vs. 26 [68.4%], *p* = 0.94), code activation rate (51.3% vs. 44.7%, *p* = 0.73), initial NIHSS score (2 [1–6] vs. 2 [1–5], *p* = 0.95), or mRS score at 3 months (1 [0–2.5] vs. 1 [0–1], *p* = 0.34) between the time periods (Table 2).

In the minor PCS sub-analysis, there was no significant difference in time to door (122 ± 67 vs. 142 ± 70 min, *p* = 0.16) and ED door to neurology referral time (80 ± 170 vs. 120 ± 108 min, *p* = 017) between two periods, but the code activation rate was significantly higher in period 1 (58.5% vs. 27.9%, *p* = 0.005). Also, there was no significant difference in functional outcome, as indicated by the mRS score at 3 months, between the two groups (1 [0–1] vs. 0 [0–1], *p* = 0.09) (Table 2).

In the non–AVS/AIS group, there was no significant between-period difference in patient age (67 ± 13 years vs. 68 ± 12 years, *p* = 0.76), sex distribution (male, 41 [54.7%] vs. 48 [63.2%], *p* = 0.37), initial NIHSS score (5 [2–15] vs. 6 [3–12], *p* = 0.35), or mRS score at 3 months (1 [1–4] vs. 1 [0–4], *p* = 0.33). However, a high code activation rate was observed in period 1 in the non-AVS/AIS group (84.0% vs. 67.1%, *p* = 0.03) (Supplementary Table 1).

There was no statistical difference between dizziness classification and stroke lesion according to period. When stroke lesions were compared according to AVS/AIS regardless of period and stroke severity, midbrain (18.5% vs. 31.7%, *p* = 0.05) and PICA lesions (20.5% vs. 41.6%, *p* = 0.001) were more frequently involved in PCS with AVS/AIS.

### Clinical Significance of AVS/AIS in Patients With Minor PCS

Compared to the non-AVS/AIS subgroup, patients with AVS/AIS in the group with minor PCS (Table 3) revealed a lower rate of unfavorable outcome (mRS score ≥2 or END; 27.5% vs. 6.7%, *p* = 0.02). According to the mRS score at 3 months, there was no significant between-group difference in functional outcome (1 [0–1] vs. 0 [0–1], *p* = 0.16). In the AVS/AIS group, there was a significantly lower code activation rate (62.8% vs. 24.4%, *p* < 0.001) and a significantly longer ED door to neurology department referral time (51 ± 72 min vs. 152 ± 186 min, *p* = 0.001). There was no significant between-group difference in the frequency of focal neurological symptoms, such as dysarthria and facial palsy, or vertebrobasilar insufficiency confirmed by CT. There was a significantly higher rate of accompanied central oculomotor signs in patients with AVS (2.0% vs. 17.8%, *p* = 0.02) (Table 3).

**Table 3:** Demographics and clinical characteristics of patients with minor posterior circulation stroke.

	**None-AVS/AIS**	**AVS/AIS**	***p*-value**
	**(*n =* 51)**	**(*n =* 45)**	
Age (years)	63 ± 10	63 ± 14	0.90
Onset to visit time (min)	139 ± 74	123 ± 63	0.26
Door to neurology department	51 ± 72	152 ± 186	0.001
referral time (min)			
Code activation, *n* (%)	32 (62.8%)	11 (24.4%)	<0.001
Reperfusion therapy, *n* (%)	2 (3.9%)	1 (2.2%)	>0.99
Initial NIHSS, median	2 [1–3]	1 [0–2]	0.04
3 month mRS, median	1 [0–1]	0 [0–1]	0.16
3 month mRS 0–1, *n* (%)	43 (84.3%)	43 (95.6%)	0.14
3 month mRS 0–2, *n* (%)	45 (88.2%)	43 (95.6%)	0.36
END, *n* (%)	9 (17.7%)	1 (2.2%)	0.03
Unfavorable outcome, *n* (%)	14 (27.5%)	3 (6.7%)	0.02
Dysarthria, *n* (%)	29 (56.9%)	17 (37.8%)	0.10
Facial palsy, *n* (%)	11 (21.6%)	9 (20.0%)	>0.99
Central oculomotor sign, *n* (%)	1 (2.0%)	8 (17.8%)	0.02
Vertebrobasilar steno-occlusion, *n* (%)	17 (33.3%)	7 (15.6%)	0.09
TOAST classification, *n* (%)			0.13
LAA	16 (31.4%)	16 (35.6%)	
CAE	5 (9.8%)	7 (15.6%)	
SVO	22 (43.10%)	10 (22.2%)	
OD	1 (2.0%)	5 (11.1%)	
UN	7 (13.7%)	7 (15.6%)	

*AIS, acute imbalance syndrome; AVS, acute vestibular syndrome; CAE, cardioembolism; END, early neurological deterioration; LAA, large artery atherosclerosis; OD, stroke of other determined etiology; SVO, small vessel occlusion; TOAST classification; UN, stroke of undetermined etiology*.

In the multivariable analysis, AVS/AIS (OR 7.8, CI 1.5–39.4; *p* = 0.01), facial palsy (OR 6.2, CI 1.1–35.4; *p* = 0.04), and vertebrobasilar steno-occlusion (OR 19.4, CI 1.9–194.6; *p* = 0.01) were associated with good outcomes. Dysarthria was negatively correlated (OR 0.1, CI 0.01–0.5; *p* = 0.005), with age and code activation rate as covariates (Table 4).

**Table 4:** Clinical predictors of a good outcome in patients with minor posterior circulation stroke.

**Parameter**	**Multivariate analysis**	
	**OR (95% CI)**	***p*-value**
AIS/AVS	7.8 [1.5–39.4]	0.01
Age	1.0 [0.9–1.1]	0.74
Code activation	0.9 [0.2–3.8]	0.91
Dysarthria	0.1 [0.01–0.5]	0.005
Facial palsy	6.2 [1.1–35.4]	0.04
Vertebrobasilar steno-occlusion	19.4 [1.9–194.6]	0.01

*AIS, acute imbalance syndrome; AVS, acute vestibular syndrome; CI, confidence interval; OR, odds ratio*.

### Clinical Significance of AVS/AIS and Central Oculomotor Signs in Patients With Major PCS

In the group with major PCS, AVS/AIS was frequently prodromal (23/32, 71.8%). Accordingly, patients with major PCS were divided into three groups: non-AVS/AIS group (*n* = 100, 75.8%), those with AVS/AIS (*n* = 9, 6.8%), and those with prodromal AVS/AIS (*n* = 23, 17.4%) (Table 5). There was no significant difference in patient age (70 ± 13 years vs. 68 ± 15 years vs. 63 ± 14 years, *p* = 0.18) or onset to visit time **(**108 ± 63 min vs. 144 ± 57 min vs. 103 ± 60 min, *p* = 0.20) between the three groups. However, the ED door to neurology department referral time was shorter in non-AVS/AIS group and prodromal AVS/AIS group when comparing with AVS/AIS group (non-AVS/AIS vs. AVS/AIS vs. prodromal AVS/AIS, listed in this order here-on; 29 ± 50 vs. 143 ± 108 vs. 35 ± 64 min, *p* = 0.02). In the AVS/AIS group, there was a lower code activation rate compared to non-AVS/AIS group, and prodromal AVS/AIS group (82.0% vs. 55.6% vs. 91.3%, *p* = 0.06). Central oculomotor signs were significantly more common in the AVS/AIS and prodromal AVS/AIS groups than in the non-AVS/AIS group (3.0% vs. 22.2% vs. 43.8%, *p* < 0.001). When etiology was analyzed by TOAST classification, large artery atherosclerosis was significantly more common in the group with prodromal AVS/AIS than in the other groups (32.0% vs. 11.1% vs. 60.9%, *p* = 0.003) and CAE was less common in the prodromal AVS/AIS group than in the other groups (28.0% vs. 44.4% vs. 8.7%, *p* = 0.003). While the rate of reperfusion treatment was highest in the prodromal AVS/AIS group, it did not reach clinical significance (47.0% vs. 33.3% vs. 60.9%, *p* = 0.32). There was no significant difference in functional outcome, as indicated by the mRS score at 3 months, between the three groups (2 [1–5] vs. 2 [1–3] vs. 2 [1–5], *p* = 0.53) (Table 5). In multivariable analysis, there was no association of presence of AVS/AIS with a good outcome (*p* = 0.71) when age, mental status, hemiparesis, reperfusion treatments, and vertebrobasilar steno-occlusion were incorporated as covariates (Table 6).

**Table 5:** Demographics and clinical characteristics of patients with major posterior circulation stroke.

	**None-AVS/AIS**	**AVS/AIS**	**Prodromal**	***p*-value**
	**(*n =* 100)**	**(*n =* 9)**	**(*n =* 23)**	
Age (years)	70 ± 13	68 ± 15	63 ± 14	0.18
Onset to visit time (min)	108 ± 63	144 ± 57	103 ± 60	0.20
Door to neurology department referral time (min)	29 ± 50	143 ± 108	35 ± 64	0.02
Code activation, *n* (%)	82 (82.0%)	5 (55.6%)	21 (91.3%)	0.06
Reperfusion therapy, *n* (%)	47 (47.0%)	3 (33.3%)	14 (60.9%)	0.32
Door to needle time (min)	61 ± 38	128	63 ± 31	–
Door to groin puncture time (min)	118 ± 30	467 ± 433	123 ± 45	0.63
Initial NIHSS, median	10 [5–18.25]	5 [4–9]	8 [3–20.5]	0.09
3 month mRS, median	2 [1–5]	2 [1–3]	2 [1–5]	0.53
3 month mRS 0–2, *n* (%)	51 (51.0%)	5 (55.6%)	12 (52.2%)	0.96
END, *n* (%)	20 (20.0%)	1 (11.1%)	8 (34.8%)	0.22
Dysarthria, *n* (%)	86 (86.0%)	7 (77.8%)	16 (69.6%)	0.16
Facial palsy, *n* (%)	64 (64.0%)	7 (77.8%)	18 (78.3%)	0.33
Central oculomotor sign, *n* (%)	2 (3.0%)	2 (22.2%)	7 (43.8%)	<0.001
Decreased mental alertness, *n* (%)	52 (52.0%)	1 (11.1%)	12 (52.2%)	0.06
Hemiparesis (2 or more), *n* (%)	86 (86.0%)	6 (66.7%)	17 (73.9%)	0.17
Vertebrobasilar steno-occlusion, *n* (%)	60 (60.0%)	3 (33.3%)	15 (65.2%)	0.24
TOAST classification, *n* (%)				0.003
LAA	32 (32.0%)	1 (11.1%)	14 (60.9%)	
CAE	28 (28.0%)	4 (44.4%)	2 (8.7%)	
SVO	21 (21.0%)	2 (22.2%)	4 (17.4%)	
OD	1 (1.0%)	1 (11.1%)	3 (13.0%)	
UN	18 (18.0%)	1 (11.1%)	0 (0.0%)	

*AIS, acute imbalance syndrome; AVS, acute vestibular syndrome; CAE, cardioembolism; END, early neurological deterioration; LAA, large artery atherosclerosis; NIHSS, National Institutes of Health Stroke Scale; mRS, modified Rankin Scale; OD, stroke of other determined etiology; SVO, small vessel occlusion; TOAST classification; UN, stroke of undetermined etiology*.

**Table 6:** Clinical predictors of a good outcome in patients with major posterior circulation stroke.

**Parameter**	**Multivariate analysis**	
	**OR (95% CI)**	***p*-value**
Presence of AVS/AIS		0.71
None	Reference	
AVS/AIS	0.5 [0.1–2.6]	0.43
Prodromal	0.8 [0.3–2.4]	0.70
Age	0.96 [0.9–0.99]	0.01
Decreased mental alertness	0.4 [0.2–0.98]	0.04
Hemiparesis	0.3 [0.1–1.04]	0.06
EVT	0.5 [0.2–1.4]	0.17
Vertebrobasilar steno-occlusion	0.9 [0.3−2.2]	0.73

*AIS, acute imbalance syndrome; AVS, acute vestibular syndrome; EVT, endovascular treatment*.

The time from the onset of AVS/AIS to the major neurological deficits in patients with prodromal AVS/AIS was median 630 (Interquartile range: 56–1,440) min. Regarding their temporal profile, among 23 patients with prodromal AVS/AIS, 9 (39.1%) patients worsened with occurrence of major deficits within 3 h, 18 (78.2%) patients worsened within 24 h, and 20 patients worsened within 3 days (87.0%) with prodromal AVS/AIS. Regarding prior medical care before deterioration, 12 (52.2%) experienced deterioration before seeking medical care, and 1 (4.3%) deteriorated while presenting to a clinic for AVS/AIS symptoms. Eleven patients (47.8%) patients deteriorated after emergency medical care, in which 8 patients deteriorated while admitted to primary hospitals due to vertigo, 1 presented to our ED due to vertigo but deteriorated before clinical assessment, and 1 patient presented to our ED for AVS 3 days before the stroke event, but was discharged.

**Figure 1:**
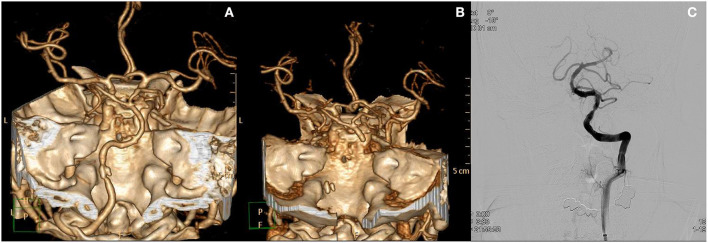
Case of a patient who presented with prodromal acute vestibular syndrome. **(A)** Initial CT angiography revealed focal calcified plaque at the left distal vertebral artery with stenosis. The patient was discharged from the ED. Three days later, the patient revisited the ED with newly onset major neurological deficits. **(B)** CT angiography at that time show occlusion from the left vertebral artery to the distal basilar artery. **(C)** Recanalization of basilar and vertebral arteries after endovascular treatment.

The following is an example of the last patient who presented with prodromal AVS (Figure 1). A 50 year-old man with a past history of schizophrenia visited the ER with a 6 day history of dizziness, headache, and vomiting. The patient did not show any focal neurological deficits. CT angiography was performed in the ER and revealed calcified plaque with stenosis in the dominant left vertebral artery. The patient did not agree to further evaluation and management and was discharged. Three days later, the patient revisited the ER with central oculomotor signs, dysarthria, facial palsy, and an NIHSS score of 13. CT angiography showed occlusion of the vertebral and basilar arteries, and mechanical thrombectomy was needed for arterial reperfusion.

## Discussion

In this study, we failed to show that differences in how AVS/AIS is addressed in the stroke thrombolysis code would influence the quality of hyperacute treatment and affect clinical outcomes. In the minor PCS group, AVS/AIS was associated with good outcomes. In the major PCS group, while AVS/AIS was not associated with clinical outcomes, a clinically significant group of PCS patients presenting with prodromal vertigo could be identified.

Our results did not show differences in PCS stroke outcomes according to changes in institutional thrombolysis code despite actual changes in the percentage of patients referred to the neurology department for possible thrombolysis. The decreases in code activation was not associated with poorer functional outcomes, but it was also not associated with decreased rates of reperfusion therapy. There may be two explanations. First, even with changes in thrombolysis code, this change may have not been sufficient to lead to a clinically significant change in medical practice, especially because AVS/AIS patients were excluded from thrombolysis code, not the other way around. If clinical differences in outcomes were to occur between the two time periods, it would most likely be led by a larger number of minor PCS patients presenting with AVS/AIS, referred early to neurologists, and treated with hyperacute stroke treatments such as IVT or EVT. However, differences in thrombolysis code was not associated with changes in rates of reperfusion therapy. Scarce scientific evidence for reperfusion therapy in AVS/AIS seemed to limit radical changes regarding management of this population. Second, while our study results do not directly address the issue of IVT in AVS/AIS, which is still uncertain (13, 31), it may present indirect supporting evidence that treatment effects of aggressive screening of isolated AVS/AIS patients for thrombolysis may not be cost-effective. However, while the current study judged the cost-effectiveness of thrombolysis protocol based on the rate of time metrics, reperfusion treatments, and clinical outcome, it may be reasonable to also evaluate the rate of false-positive code activations and false-negative code activations in future studies. As this study included only patients with PCS, according data could not be shown in this study. We hope to address this issue in future studies.

Our study results do not claim against reperfusion therapy in PCS patients with AVS/AIS. While isolated AVS/AIS is often not considered disabling to justify IVT (31), a recent study involving a small number of PCS patients presenting with dizziness found IVT to be associated with salvage of brain tissues (13), which is encouraging. More sensitive neurological scales may be needed for prompt identification of PCS patients presenting with AVS/AIS, and to include them for future thrombolysis trials. This is evidenced by longer referral time for the AVS/AIS group compared to the non-AVS/AIS group in both minor and major PCS group. Prompt detection of PCS patients may be limited by the NIHSS, which is not known to be very sensitive in PCS (32), and under-estimates stroke severity and following functional neurological deficits (15). A previous study evaluating IVT in isolated AVS/AIS also faced similar issues, as decision for IVT was largely led by focal neurological deficits previously known to be disabling, and higher NIHSS stroke scales (33). In this regard, the use of extended clinical scales such as the e-NIHSS, which adds specific elements in existing items to explore signs or symptoms of posterior circulation stroke, may improve the sensitivity of PCS detection, and may aid future studies regarding reperfusion therapy in PCS (32).

Our data shows the importance of identifying prodromal AVS/AIS, which was the dominant AVS/AIS pattern in major PCS. In this group, large artery atherosclerosis was more common, and over half the patients underwent reperfusion therapy. They also experienced END in 1/3 of the patients. In addition, the time interval from AVS/AIS to major deficits were within 3 days for the majority of the patients. It will be important to identify prodromal AVS/AIS patients in the prodromal stage without major deficits. To achieve this goal, careful description of vertigo characteristics in this prodromal group must be performed. A recent literature described that transient vestibular symptoms preceding PCS occurred in 12% during the 3 previous 3 months, which is usually vertigo with or without imbalance, with durations usually seconds to minutes (34). Similar studies should be further performed to identify the population of prodromal vertigo. The HINTS (Head-Impulse, Nystagmus, Test-of-Skew) may be helpful to differentiate prodromal vertigo. The HINTS has been extensively verified for its ability to distinguish central vertigo rather than peripheral vertigo among patients with AVS (35, 36). The ability of HINTS to identify patients at high risk for neurological deterioration needs to be confirmed in future studies. Also, presence of upbeat or downbeat nystagmus, which indicates vestibular imbalance in the pitch plane and bilateral vestibular structure involvement, may be signs that suggest global hypoperfusion and impending neurological aggravation (37). Imaging modalities for patient screening in the emergency department should also focus in identification of this potentially critical population. The presence of vertebrobasilar steno-occlusion in over half of this population emphasizes the importance of arterial imaging in acute vertigo and imbalance. This may be further aided by perfusion imaging (38), which may detect brain tissue that may be salvaged with hyperacute stroke treatments (13). Due to the high rates of large artery atherosclerosis in this population, dual antiplatelets and high dose statins to stabilize atherosclerotic plaque (39) may be the preferred treatment in this population. In the meantime, the clinical characteristics of this potentially grave population identified in this study may provide evidence for the length of hospital admission for those patients in which central vertigo is suspected, but not yet confirmed by imaging findings.

In patients with minor PCS, AVS/AIS was associated with lower NIHSS scores, lower rates of END, and better outcomes. There may be two possible explanations. First, compared to PCS patients with other minor symptoms, disabling neurological deficits may be less likely accompanied, or less likely to occur in the treatment course in PCS patients with AVS/AIS. Sustained vertigo with direction specific falls is associated with lesions of the vestibular nuclei and vestibular cerebellum causing vestibular toner imbalance in the yaw plane (40). Commonly accompanied oculomotor findings such as ocular tilt reaction or lateropulsion is caused by vestibular imbalance in the roll plane. Such vestibular asymmetry in the yaw or roll plane point to a unilateral disease of the brainstem, and usually do not indicate a progressive infratentorial stroke (41). Furthermore, corticospinal tract is located in the medial brainstem, while lesions causing isolated dizziness and vertigo is usually localized to the lateral brainstem (37). The finding that dysarthria, a sign of corticobular and corticospinal tract involvement (42), was associated with poor clinical outcomes, is supportive of this view. Second, vertigo, imbalance itself as a disabling symptom may be less significant, as vestibular tone imbalance due to central lesions are known to resolve within 2–4 weeks as a compensatory process (43). The cerebellum takes role in this compensatory mechanism within weeks, unless the patients have lesions in specific regions within the cerebellar hemispheres which might hinder compensatory processes (44, 45). However, care should be noted in interpretation of the current findings because previous studies have found non-lacunar mechanisms in half of stroke patients presenting with AVS (46), and heterogenous stroke etiologies are also seen in our study.

In our study, vertebrobasilar steno-occlusion and facial palsy were also identified as a good prognostic factor in patients with minor PCS. In the minor deficit subgroup, most of the vertebrobasilar steno-occlusion would have been chronic, and collateral vessels may have been well-developed due to longstanding hypoperfusion (47), contributing to better outcomes. The facial motor nucleus is located in the lateral brainstem (48), rather than the medial brainstem in which the corticospinal tract is located, and which would be a key determinant of functional outcomes if involved by a stroke lesion. These findings and corresponding mechanisms need to be validated in future studies.

Previous literature had classified acute AVS patients as isolated AVS and non-isolated AVS (49). It is clinically important to differentiate isolated vascular vertigo (37) from benign causes of AVS such as vestibular neuritis. However, the main focus of the current study was influence of vertigo on thrombolysis, and we chose to classify their symptoms by presence of AVS/AIS, and also by clinical severity as major and minor deficits (13). Furthermore, another important focus of our study was whether changes in thrombolysis code will cause delays in acute stroke treatment. This would usually be more dominant in patients without obvious major neurological deficits, and caused by delayed neurologist referral by emergency physicians due to uncertainty in diagnosis, and neurologists pondering over therapeutic benefits. This is another reason we classified their symptoms into major and minor deficits.

Our study has several limitations. First, as this analysis was performed through analysis of a stroke database, worsened clinical outcomes due to misdiagnosis of central vertigo may not be appropriately represented. However, such patients are at least partly represented by the prodromal vertigo group in this study, and we believe that our study results is distinguished from analysis of AVS/AIS patients because the clinical course of all PCS patients is described. Second, due to the retrospective observational design the classification of dizziness information regarding central oculomotor signs may be inaccurate. However, as all patients were admitted with multiple neurological exams performed by stroke staff, neurology residents, and stroke nurse in the stroke unit, it is unlikely that symptoms were omitted to a large degree. Third, there still remains a gap between minor AVS/AIS group and prodromal vertigo group, as END was infrequent in minor AVS/AIS. Thus, in future studies, risk factors of END in minor AVS/AIS should be identified, such as incomplete occlusions or distal basilar involvements that have predicted END in vertebrobasilar occlusions (50, 51).

In conclusion, an emphasis on AVS/AIS in the stroke thrombolysis code was not associated with higher thrombolysis rates nor with differences in clinical outcomes. In PCS patients presenting with AVS/AIS without severe focal neurological deficits, the clinical course seems to be usually benign. However, a substantial percentage of patients presented with prodromal AVS/AIS in major PCS, and care should be taken to identify this subgroup in the prodromal phase.

## Data Availability Statement

The raw data supporting the conclusions of this article will be made available by the authors, without undue reservation.

## Ethics Statement

The studies involving human participants were reviewed and approved by Ethics Committee and Institutional Review Board of Ajou University Hospital. Written informed consent for participation was not required for this study in accordance with the national legislation and the institutional requirements.

## Author Contributions

MK: data interpretation, drafted the work, revised the draft critically for important intellectual content, and approved the final version of the paper. SP, SL, JL, and JH: data interpretation, revised the draft critically for important intellectual content, and approved the final version of the paper. S-JL: conceptualization and supervision of the study, data interpretation, revised the draft critically for important intellectual content, and approved the final version of the paper.

## Funding

This work was supported by the Basic Science Research Program through the National Research Foundation of Korea (NRF) funded by the Ministry of Education (NRF-2021R1I1A1A01048331; S-JL).

## Conflict of Interest

The authors declare that the research was conducted in the absence of any commercial or financial relationships that could be construed as a potential conflict of interest.

## Publisher's Note

All claims expressed in this article are solely those of the authors and do not necessarily represent those of their affiliated organizations, or those of the publisher, the editors and the reviewers. Any product that may be evaluated in this article, or claim that may be made by its manufacturer, is not guaranteed or endorsed by the publisher.
