# Supplementary material for: Significance of Vertigo, Imbalance, and Other Minor Symptoms in Hyperacute Treatment of Posterior Circulation Stroke
Source: Front Neurol. 2022 May 16;13:845707. doi: 10.3389/fneur.2022.845707 (PMC9150563; doi:10.3389/fneur.2022.845707)
Supplement: Supplementary file 1 [file Table_1.docx]

**Significance of vertigo, imbalance, and other minor symptoms in hyperacute treatment of posterior circulation stroke**

**Supplementary Table 1.** Comparison between two periods for patients with posterior circulation stroke that presented without acute vestibular syndrome or acute imbalance syndrome.

|  | **Period 1**  **(n=75)** | **Period 2**  **(n=76)** | **p-value** |
| --- | --- | --- | --- |
| Age (years) | 67 ± 13 | 68 ± 12 | 0.76 |
| Sex (male, %) | 41 (54.7%) | 48 (68.2%) | 0.37 |
| Onset to visit time (min) | 110 ± 66 | 127 ± 70 | 0.01 |
| Door to neurology department  referral time (min) | 24 ± 41 | 48 ± 71 | 0.13 |
| Code activation, n (%) | 63 (84.0%) | 51 (67.1%) | **0.03** |
| IVT, n (%) | 19 (25.3%) | 15 (19.7%) | 0.53 |
| Door to needle time (min) | 47.2 ± 11.2 | 75.9 ± 51.4 | 0.06 |
| EVT, n (%) | 19 (25.3%) | 11 (14.5%) | 0.14 |
| Door to groin puncture time (min) | 114 ± 32 | 122 ± 26 | 0.48 |
| Initial NIHSS, median | 5 [2–15] | 6 [3–12] | 0.35 |
| 3 months mRS, median | 1 [1–4] | 1 [0–4] | 0.33 |
| 3 months mRS 0–1, n (%) | 40 (53.3%) | 44 (57.9%) | 0.69 |
| 3 months mRS 0–2, n (%) | 45 (60%) | 51 (67.1%) | 0.46 |
| END, n (%) | 16 (21.3%) | 13 (17.1%) | 0.65 |
